# Two New Flavonol Glycosides from *Sarcopyramis bodinieri* var. *delicate*

**DOI:** 10.3390/molecules13061399

**Published:** 2008-06-19

**Authors:** Xiu Min Wang, Chun Peng Wan, Shou Ran Zhou, Yan Qiu

**Affiliations:** 1 Department of Pharmacy, School of Medical, Xiamen University, Xiamen 361005, People′s Republic of China; E-mail: wangxm@xmu.edu.cn; 2 Jiangxi University of Traditional Chinese Medicine, Nanchang 330006, People′s Republic of China; E-mails: lemonwan@126.com (Wan); 9x8f@163.com (Zhou)

**Keywords:** *Sarcopyramis bodinieri* var*. delicate*, flavonol glycoside

## Abstract

Detailed chemical investigation of the herb *Sarcopyramis bodinieri* var*. delicate* resulted in the isolation of two new flavonol glycosides, namely, isorhamnetin-3-*O*-(6′′-*O*-*E*-feruloyl)-*β*-D-glucopyranoside (**1**) and isorhamnetin-3-*O*-(6′′-*O*-*E*-feruloyl)-*β*-D-galactopyranoside (**2**). In addition, four known compounds, quercetin-3-*O*-(6′′-acetyl)-*β*-D-glucopyranoside (**3**), isorhamnetin-3-*O*-(6′′-acetyl)-*β*-D-glucopyranoside (**4**), quercetin-3-*O*-(6′′-*O*-*E*-*p*-coumaroyl)-*β*-D-glucopyranoside (**5**), and isorhamnetin-3-*O*-(6′′-*O*-*E*-*p*-coumaroyl)-*β*-D-glucopyranoside (**6**) were obtained. The structures of the new isolates were determined by extensive spectroscopic analysis.

## Introduction

Members of the Melastomataceae family are widespread in many regions of the world and particularly in tropical and subtropical regions, mainly in South America and South China. Many species of this family are known by their different use in folk medicine as antioxidant [1], antihypertensive [2], antihyperglycemic [3], hemostatic [4] and antihepatitis drugs [5]. Antiinflammatory [4], antimicrobial [6] and cytotoxic effects [1] have also been investigated. Cumulative phytochemical studies of Melastomataceous plants have indicated an abundance of tannins [7], polyphenols [7], flavonoids [8], fatty acids, steroids, and free triterpenoids [9]. The great variety of natural compounds found in this family, as well as their pharmacological properties prompted us to study the chemical constituents and bioactivities of *Sarcopyramis bodinieri* var*. delicate*, an endemic and chemically uninvestigated plant distributed widely in South China.

**Figure 1:**
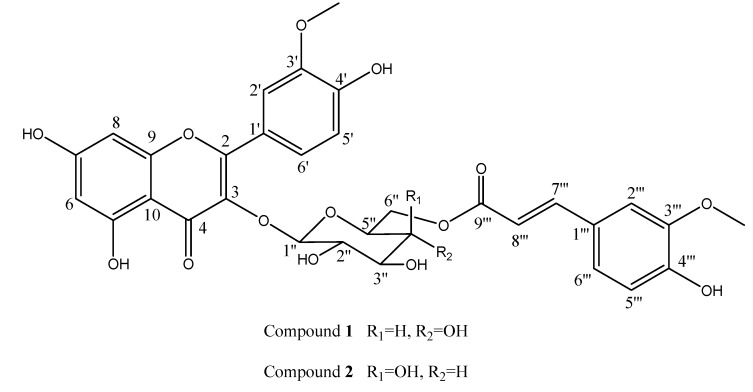
The structures of new compounds **1** and **2**.

As a rare species, *Sarcopyramis bodinieri* var*. delicate* was widely used as hepatoprotective drug in Fujian province, China. The water extract of this dried herb could reduce aminotransferase and cure choloplania and hepatoma. Detailed fractionation led to the isolation of two new flavonol glycosides, namely, isorhamnetin-3-*O*-(6′′-*O*-*E*-feruloyl)-*β*-D-glucopyranoside (**1**) and isorhamnetin-3-*O*-(6′′-*O*-*E*-feruloyl)-*β*-D-galactopyranoside (**2**), along with four known compounds. The structures of the new isolates were determined by extensive spectroscopic analysis.(Figure 1)

## Results and Discussion

The known compounds **3-6** corresponded to quercetin-3-*O*-(6′′-acetyl)-*β*-D-glucopyranoside (**3**) [10], isorhamnetin-3-*O*-(6′′-acetyl)-*β*-D-glucopyranoside (**4**) [11], quercetin-3-*O*-(6′′-*O*-*E*-*p*-coumaroyl)-*β*-D-glucopyranoside (**5**) [12], and isorhamnetin-3-*O*-(6′′-*O*-*E*-*p*-coumaroyl)-*β*-D-glucopyranoside (**6**) [13,14]. The structures of these known flavonol glycosides were identified on the basis of extensive spectroscopic data analysis and by comparison of their spectral data with those reported in the literature.

**Figure 2:**
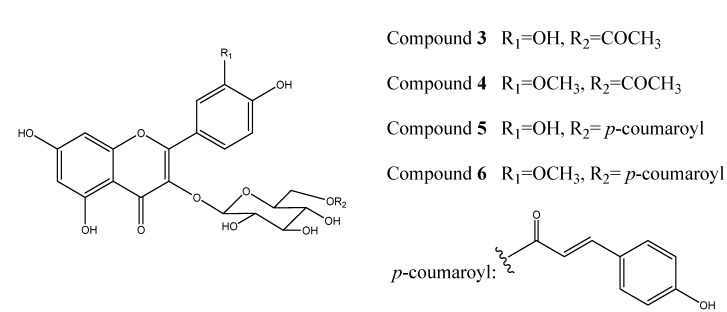
The structures of compounds **3-6**.

Compound **1** was isolated as a yellow powder. The molecular formula C_32_H_30_O_15_ was suggested by a mass spectrum with a [M+Na]^+^ peak at *m/z* 677, further confirmed by the adduct ions: 693 [M+K]^+^, and 1331 [2M+Na]^+^, combined with the ^13^C-NMR and DEPT spectra. The IR spectrum of compound **1** revealed the aliphatic and aromatic hydroxyl signals at 3200-3550 cm^-1^. A conjugated carbonyl group (1656 cm^-1^) and an additional *α,β*-unsaturated carbonyl ester group (1724 cm^-1^) were observed in the same region. The absorptions at 3310 and 1057 cm^-1^ indicated the presence of a glycosidic moiety [15].

The ^1^H-NMR spectrum confirmed many of the above features and revealed a set of isorhamnetin signals, a feruloyl group and a glucopyranose moiety. The presence of isorhamnetin was suggested by the following signals: two doublets at *δ*_H_ 6.14 (d, *J* = 1.8 Hz, H-6) and 6.37 (d, *J* = 1.8 Hz, H-8); an ABX spin system due to the aromatic ring at *δ*_H_ 6.90 (d, *J* = 8.6 Hz, H-5′), 7.52 (dd, *J* = 8.6, 1.9 Hz, H-6′) and 7.87 (d, *J* = 1.9 Hz, H-2′); a methoxyl group singlet at *δ*_H_ 3.86 (s, 3′-OMe) with a HMBC correlation with *δ*_C_ 147.1 (C-3′) [16]. In the ^1^H-NMR spectrum another set of ABX-type aromatic proton signals at *δ*_H_ 6.75 (d, *J* = 8.1 Hz, H-5′′′), 6.88 (dd, *J* = 8.1, 1.2 Hz, H-6′′′) and 7.20 (d, *J* = 1.2 Hz, H-2′′′) were observed, together with an additional HMBC crosspeak between *δ*_H_ 3.82 (s, 3′′′-OMe) and *δ*_C_ 147.9 (C-3′′′). Two olefinic protons with a *trans* coupling constant (*J* = 15.8 Hz) at *δ*_H_ 6.22 (d, H-8′′′), 7.34 (d, H-7′′′), which confirmed the presence of an *α,β*-unsaturated carbonyl ester group. The feruloyl structure was then deduced from the HMBC correlation from *δ*_H_ 6.22 (H-8′′′) to *δ*_C_ 126.4 (C-1′′′) and the crosspeaks between H-2′′′/C-7′′′ and H-6′′′/C-7′′′. Detailed analyses of the ^1^H- (*δ* 5.53, d, *J* = 7.8 Hz, H-1′′) and ^13^C-NMR (*δ* 102.8, 77.8, 76.1, 76.0, 71.8, 64.2) suggested glucopyranose as the sugar moiety. A downfield shift of C-6′′ was from *δ*_C_ 61.8 to 64.2, and an upfield shift of C-5′′ was from *δ*_C_ 76.8 to 76.0, which were in accordance with the acylation of C-6′′ of the glucose moiety [17]. Moreover, the downfield shift of H_2_-6′′ to 4.11 (dd, *J* = 6.8, 11.5 Hz) and 4.19 (dd, *J* = 2.1, 11.5 Hz) further confirmed the presence of a C-6′′ feruloyl in compound **1** [18]. Consequently, the structure of compound **1** was established as isorhamnetin-3-*O*-(6′′-*O*-*E*-feruloyl)-*β*-D-glucopyranoside.

Compound **2**, a yellow powder, shared the same molecular formula C_32_H_30_O_15_ with **1**, according to the [M+Na]^+^ peak at *m/z* 677 and [M+K]^+^ peak at *m/z* 693. Moreover, its NMR data is very similar to those of **1**. Detailed comparison of the ^13^C-NMR and HMQC spectra between the two compounds indicated that the major difference was in the glycoside moiety. The carbon signals at *δ*_C_ 103.4 (C-1′′), 71.2 (C-2′′), 74.6 (C-3′′), 69.5 (C-4′′), 74.3 (C-5′′), and 62.9 (C-6′′) revealed a galactopyranoside moiety [16]. The structure of compound **2** was therefore assigned as isorhamnetin-3-*O*-(6′′-*O*-*E*-feruloyl)-*β*-D-galactopyranoside.

**Table 1:** The ^1^H and ^13^C-NMR data of compounds **1** and **2** (DMSO-*d*_6_).

No.	Compound **1**		Compound **2**	
	H (*J*_Hz_)	C	H (*J*_Hz_)	C
2		157.6		157.5
3		134.3		134.3
4		178.6		178.5
5		162.3		162.2
6	6.14 d (1.8)	99.6	6.15 d (1.8)	99.5
7		165.1		165.1
8	6.37 d (1.8)	94.9	6.37 d (1.8)	94.7
9		157.6		157.5
10		104.9		104.9
1′		122.3		122.2
2′	7.87 d (1.9)	114.2	7.99 d (1.9)	114.5
3′		147.1		147.1
4′		150.7		150.6
5′	6.90 d (8.6)	116.1	6.89 d (8.6)	116.2
6′	7.52 dd (8.6, 1.9)	122.9	7.50 dd (8.6, 1.9)	122.9
1′′	5.53 d (7.8)	102.8	5.52 d (7.8)	103.4
2′′	3.26 m	76.1	3.20 m	71.2
3′′	3.28 m	77.8	3.48 m	74.6
4′′	3.62 m	71.8	3.70 m	69.5
5′′	3.43 m	76.0	3.74 m	74.3
6′′	4.11 dd (6.8, 11.5) 4.19 dd (2.1, 11.5)	64.2	4.12 dd (7.0, 11.8) 4.24 dd (2.1, 11.8)	62.9
1′′′		126.4		126.4
2′′′	7.20 d (1.2)	112.1	7.19 d (1.2)	111.7
3′′′		147.9		147.8
4′′′		150.3		150.4
5′′′	6.75 d (8.1)	116.4	6.78 d (8.0)	116.4
6′′′	6.88 dd (8.1, 1.2)	123.8	6.88 dd (8.0, 1.2)	124.0
7′′′	7.34 d (15.8)	145.9	7.34 d (15.8)	146.0
8′′′	6.22 d (15.8)	115.1	6.26 d (15.8)	113.8
9′′′		167.5		167.2
3′-OMe	3.86	56.9	3.87	56.8
3′′′-OMe	3.82	56.6	3.78	56.2

^1^H and ^13^C-NMR spectra were obtained at 600 and 150 MHz, respectively.

The UV spectra of the different flavonol glycosides showed an interesting phenomenon (see Table 2) according to our experiments. The substituted fraction on the glycosidic moiety could be characterized by the absorption over 300 nm. For example, the absorption maxima at 315 nm means a *p*-substituted aromatic ring in R_2_, while the absorption at 330-336 nm indicates a tri-substituted aromatic ring in the same position.

**Table 2:** The UV data for different flavonol glycosides. 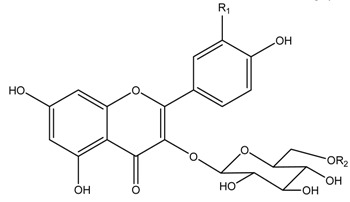

Compound		UV (MeOH) *λ*_max_	
R_1_	R_2_		
OH	H	356	258
OCH_3_	H	354	256
OH	COCH_3_	357	256
OCH_3_	COCH_3_	355	256
H	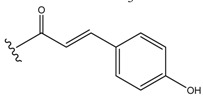	315	266
OH	the same as above	315	263
OCH_3_	the same as above	315	259
OH	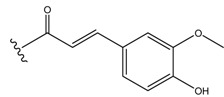	333	254
OCH_3_	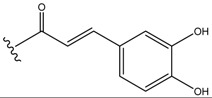	336	252

## Experimental

### General

The IR spectra were determined on a Thermo Nicolet Nexus 470 FT-IR spectrometer. Optical rotations were measured with a Perkin-Elmer 243 B polarimeter using a 1 dm microcell. The ^1^H-NMR and ^13^C-NMR spectra were recorded on a Bruker Avance-600 FT NMR spectrometer. ESI-MS were recorded on a PE Q-STAR ESI-TOF-MS/MS spectrometer. Column chromatography was carried with silica gel (200-300 mesh), and HF_254_ silica gel for TLC was obtained from Qingdao Marine Chemistry Co. Ltd., Qingdao, People′s Republic of China. ODS and Sephadex LH-20 (18-110 μm) were obtained from Pharmacia Co.

### Extraction and Isolation

The specimen of *Sarcopyramis bodinieri* var*. delicate* was collected from Fujian Province, P.R. China, in April 2007. A voucher specimen (RSC07) is deposited at the Department of Pharmacy, School of Medical, Xiamen University. The air-dried plant material (5 kg) was ground and extracted exhaustively by maceration at room temperature with EtOH-H_2_O (70:30, 20 L×3). The concentrated total extract (1.8 kg) was extracted with petroleum ether, CHCl_3_, EtOAc and *n*-BuOH, respectively. Part of the EtOAc portion (SBC, 45 g) was suspended in H_2_O (2 L), and the filter layer was then subjected to D101 macroporous adsorption resin column, eluted with an equivalent H_2_O-EtOH stepwise gradient to obtain 5 fractions. Fraction 3 (SBC-C, 13.76 g) was subjected to a Sephadex LH-20 eluted with MeOH-H_2_O (3:1) to give 6 fractions. Fr. SBC-C3 was subjected to Sephadex LH-20 column and then the repeated silica gel column to give compounds **3** (16.7 mg), **4 **(33.4 mg), **5** (18.2 mg), **6** (7.5 mg). Compounds **1** and **2** were repurified from SBC-C3C by semi-preparative reversed-phased HPLC (45% MeOH-H_2_O, YMC-Pack Pro C-18, 20×150 mm) to give 4.6 mg and 5.8 mg, respectively.

*Compound* **1**: 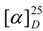 = -48.6 (*c* 0.67, MeOH); UV (MeOH) *λ*_max_ (log *ε*) 251 (4.53), 333 (3.21) nm; IR (neat) *ν*_max_ 3550, 1724, 1656, 1057 cm^-1^; for ^1^H and ^13^C-NMR see Table 1; ESI-TOF MS (*m/z*): 677 [M+Na]^+^, 693 [M+K]^+^, 1331 [2M+Na]^+^.

*Compound* **2**: 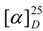 = -64.2 (*c* 0.24, MeOH); UV (MeOH) *λ*_max_ (log *ε*) 249 (4.47), 334 (3.20) nm; for ^1^H and ^13^C-NMR see Table 1; ESI-TOF MS (*m/z*): 677 [M+Na]^+^, 693 [M+K]^+^.
